# Diagnostic performance of microvascular flow imaging for noninvasive assessment of liver fibrosis in chronic liver disease

**DOI:** 10.1371/journal.pone.0322102

**Published:** 2025-06-04

**Authors:** Hae Won Yoo, Chan Jin Yang, Jeong-Ju Yoo, Young Chang, Sae Hwan Lee, Soung Won Jeong, Jae Young Jang, Gab Jin Cheon, Young Seok Kim, Hong Soo Kim, Sang Gyune Kim

**Affiliations:** 1 Department of Internal Medicine, Division of Gastroenterology and Hepatology, Soon Chun Hyang University Bucheon Hospital, Bucheon, Korea; 2 Department of Internal Medicine, Soon Chun Hyang University Seoul Hospital, Seoul, Korea; 3 Department of Internal Medicine, Soon Chun Hyang University Chunan Hospital, Chunan, Korea; 4 Department of Internal Medicine, Gangneung Asan Hospital, Gangneung, Korea; Changhua Christian Hospital, TAIWAN

## Abstract

**Background and aims:**

Chronic liver disease (CLD) represents a significant global health challenge necessitating the evaluation of liver fibrosis. This study aimed to evaluate the diagnostic performance of microvascular flow (MV-flow) imaging in evaluating liver fibrosis and compare it with other non-invasive tools.

**Methods:**

Between July 2020 and June 2022, we prospectively enrolled patients scheduled for liver biopsy, concurrently measuring MV-flow imaging, transient elastography (TE), and two-dimensional shear wave elastography (2D-SWE) as part of the assessment process. We evaluated the diagnostic performance of MV-flow imaging, 2D-SWE, and TE based on histologic staging of liver fibrosis using the area under the receiver operating characteristic curve (AUROC), and calculated the optimal cut-off value.

**Results:**

A total of 89 participants were included. Non-alcoholic fatty liver disease was the most common etiology of CLD (32.6%). The liver fibrosis stage distribution was as follows: stage 0 (11.2%), stage 1 (31.5%), stage 2 (25.8%), stage 3 (13.5%), and stage 4 (18.0%). The MV-flow scoring system’s cut-off values and AUROCs for predicting stage 2, stage 3, and cirrhosis were 2.1 (0.836), 2.5 (0.955), and 2.9 (0.942), respectively. The MV-flow scoring system’s performance in predicting advanced fibrosis (stage 3) was comparable to TE (*p* = 0.170) and 2D-SWE (*p* = 0.456). MV-flow imaging misclassified 9.0% of patients in predicting advanced fibrosis. A sequential combination of 2D-SWE and MV-flow imaging, following the specified cut-off, minimized the risk of missing advanced fibrosis to 1.2%.

**Conclusion:**

MV-flow imaging is an effective tool for predicting liver fibrosis stage. Integrating MV-flow imaging with 2D-SWE can enhance the assessment of liver fibrosis in patients with CLD.

## Introduction

Chronic liver disease (CLD) is a significant global health concern, responsible for over 2 million deaths worldwide each year, with its global burden on the rise [1]. Among individuals with CLD, the development of cirrhosis is a leading cause of morbidity and mortality [2]. Consequently, stratifying patients according to their risk of disease progression is imperative [3].

Assessing the level of fibrosis can provide crucial insights into the extent of a patient’s CLD. Liver fibrosis is characterized by the excessive accumulation of extracellular matrix proteins, including collagen, which occurs in most forms of chronic liver diseases [4]. Advanced liver fibrosis leads to cirrhosis, end-stage liver disease, and carcinoma, and is associated with significant morbidity and mortality. Therefore, accurately determining disease severity, predicting outcomes, guiding treatment decisions, and monitoring disease progression are essential [5,6].

Traditionally, liver biopsy has been considered the gold standard for estimating liver fibrosis. However, recent meta-analyses of non-invasive tests employing elastographic techniques, such as transient elastography (TE) and two-dimensional shear wave elastography (2D-SWE), have demonstrated promising accuracy [7,8]. However, its accuracy can be influenced by factors such as acute inflammation, cholestasis, cardiac dysfunction, non-fasting state, elevated portal pressure, and hepatic congestion. While there are several reliable noninvasive tests for liver fibrosis, they all have inherent limitations, demonstrating the need for an alternative approach [9].

Cirrhosis is characterized by necroinflammation and fibrogenesis, histologically presenting as dense fibrotic septa surrounding diffuse nodular formation. This results in the destruction of parenchymal tissue and a collapse of the liver’s structural integrity. Consequently, this leads to hepatic vascular architecture distortion, increased resistance to portal blood flow, and the eventual development of portal hypertension and hepatic synthetic dysfunction [10,11]. Hence, the progression of liver fibrosis is linked with significant changes in the peripheral hepatic vasculature [12]. If accurately quantified and characterized, these changes can offer valuable insights into the severity and progression of fibrosis.

Doppler ultrasound techniques, especially those measuring blood flow in small vessels at low velocities while reducing motion artifacts, such as microvascular flow (MV-flow) imaging, have been proposed as potential tools for this purpose [13,14]. Previous histological study has shown significant vascular alterations in cirrhotic livers, including distorted portal vein branches compressed by connective tissue and an increased presence of tortuous arterioles around cirrhotic nodules. These changes suggest that advanced liver fibrosis may lead to irregular vessel patterns and wider bifurcation angles in the peripheral hepatic vasculature [15].

Preliminary studies [9,16–18] utilizing Doppler technology have indicated a substantial correlation between these vascular changes and the degree of fibrosis. Nevertheless, these studies are limited in number and scope. Consequently, there is a clear need for more comprehensive research to validate these initial findings and further elucidate the precise relationship between Doppler-measurable vascular changes and liver fibrosis. Thus, we aimed to evaluate the diagnostic performance of MV-flow imaging in assessing liver fibrosis. Furthermore, we evaluated MV-flow imaging in comparison to other widely used non-invasive tools, such as TE and 2D-SWE.

## Materials and methods

### Study design and patients

Consecutive patients scheduled for a liver biopsy between July 2020 and June 2022, aged 18–70 years, were prospectively enrolled. Pertinent clinical, demographic, and laboratory data were collected on the day of the liver biopsy using a standardized protocol.

Out of the initial 96 participants, seven were excluded from the study for various reasons. Five individuals were excluded due to elevated liver enzyme levels greater than three times the upper limit of normal, a threshold commonly used in previous studies evaluating non-invasive tools for liver fibrosis. Additionally, one participant was excluded due to the presence of ascites. Following these exclusions, a total of 89 eligible participants were included in the final analysis.

This study obtained approval from the Institutional Review Board (IRB No. 2020-03-031), and informed consent, both written and verbal, was obtained from all participants prior to inclusion. The study protocol adheres to the principles outlined in the Declaration of Helsinki.

### Liver biopsy

Percutaneous liver biopsies were conducted using an automatic side-cutting core biopsy needle (ACECUT; TSK Laboratory, Tochigi, Japan). All biopsies were performed with the guidance of ultrasonography. The specimens were then preserved in formalin. Subsequently, they were stained with hematoxylin-eosin and Masson’s trichrome for histological examination. An experienced pathologist, who remained unaware of the results of non-invasive assessments, meticulously analyzed the liver biopsy samples. The histologic scoring system followed the Batts-Ludwig Staging System, categorizing fibrosis as follows: stage 0, no fibrosis; stage 1, portal fibrosis; Stage 2, periportal fibrosis; Stage 3, architecture distortion without cirrhosis; and Stage 4, cirrhosis [19].

### Microvascular flow measurement

**Fig 1:**
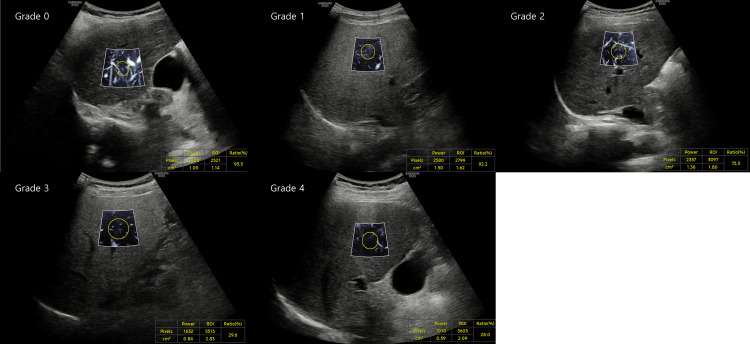
Measurements of microvascular flow (MV-flow) imaging for (a) Grade 0, (b) Grade 1, (c) Grade 2, (d) Grade 3, and (e) Grade 4.

MV-flow measurements (Fig 1) were conducted using the RS85 ultrasound device (Samsung Medison, Seoul, Korea). We performed a qualitative assessment of the vascular tree, focusing on factors such as vessel tortuosity and blunting, as detailed in Table 1. For each patient with chronic liver disease, three images were obtained. When measuring MV flow, the ultrasound probe was positioned perpendicularly. Measurements were taken at the S5 location, and images were standardized to a depth of 2–5 cm. MV-flow imaging was categorized into four stages, ranging from 0 to 4, for each image. The average score from these assessments was utilized in the subsequent analysis. The percentage of MV flow was determined by the examiner setting the size of the region of interest (ROI). Once the scan was performed at the appropriate location, the values were automatically measured and recorded.

**Table 1:** Microvascular tree grading and scoring.

					Score
**Normal (Grade 0)**					**0**
**Thinning of distal vessels (Grade 1)**	**+**				**1**
**Tortuosity in distal vessels (Grade 2)**	**+**	**+**			**2**
**Blunting of small vessels (Grade 3)**	**+**	**+**	**+**		**3**
**Blunting of large vessels (Grade 4)**	**+**	**+**	**+**	**+**	**4**

### Liver stiffness measurement

Liver stiffness (LS) measurements were performed on some patients using two devices: the 2D-SWE (RS85; Samsung Medison) and the FibroScan (Echosens, Paris, France). All elastographic evaluations were conducted within a single session, under fasting conditions. Patients were placed in a supine position with their right arm fully abducted. A single operator, experienced in ultrasound and liver elastography, conducted the examinations. The right lobe of the liver was examined via an intercostal space approach. For each patient, ten valid readings were obtained, following criteria established in previous studies to ensure reliable LS measurements [20].

### Outcomes

The primary outcome was the diagnostic performance of MV-flow imaging in assessing the degree of liver fibrosis, using liver tissue examination as the reference standard. Secondary outcomes included 1) the correlation between MV-flow imaging, 2D-SWE, and TE, 2) the optimal cut-off value, accuracy, specificity, sensitivity, positive predictive value (PPV), and negative predictive value (NPV) of each non-invasive method, and 3) the assessment of whether a combination of MV-flow imaging with other tests could enhance the differentiation of advanced fibrosis (≥stage 3).

### Statistical analysis

Data were analyzed using R statistical software (The R Foundation, Austria). Descriptive statistics were expressed as mean ± standard deviation (SD), and categorical variables were presented as frequency (percentage). Correlation analyses were conducted using the Spearman test. Cohen’s κ coefficient was used to assess the interobserver agreement of the microvascular scoring system, and the intraclass correlation coefficient (ICC) was used to determine the consistency of the measurement method on a percentage of microvascular flow. The overall diagnostic accuracy of non-invasive tools was determined through receiver-operating characteristic (ROC) curve analysis, expressed as the area under the receiver-operating characteristic curve (AUROC) with a 95% confidence interval (CI). The optimal cut-off value was established by assessing sensitivity and specificity using the Youden index. Patients were classified as positive or negative based on whether their non-invasive measurement values were greater than, less than, or equal to the estimated cut-off value. Using the cut-off value, we computed both PPV and NPV, and identified misclassified patients. Additionally, we examined the effectiveness of combining non-invasive tools sequentially using these cut-off values to determine if such combinations reduced the number of misclassified patients. The DeLong test was used to compare AUROCs among non-invasive measurements, with statistical significance set at a *p*-value less than 0.05 for all tests.

## Results

### Baseline characteristics

Ninety patients initially underwent both liver biopsy and MV-flow imaging. However, MV-flow measurement failed in one patient, leaving 89 individuals for analysis. The baseline characteristics of the study participants are summarized in Table 2. The mean age of the patients was 51.2 ± 12.3 years, and 63.1% were male. The most prevalent etiology of chronic liver disease was alcoholic fatty liver disease (32.6%), followed by non-alcoholic fatty liver disease (22.5%).

**Table 2:** Patients’ characteristics.

No. of Patients	89
Age (years), Mean ± SD	51.1 ± 12.1
Gender, n (%)	
Male	56 (62.9%)
Female	33 (37.1%)
Etiology, n (%)	
HBV	12 (13.5%)
MASLD	20 (22.5%)
ALD	29 (32.6%)
HCV	0 (0%)
AIH/PBC	15 (16.9%)
Others	13 (14.6%)
Length of biopsy specimen	32.7 ± 5.0
Fibrosis Stage^†^, n (%)	
Stage 0	10 (11.2%)
Stage 1	28 (31.5%)
Stage 2	23 (25.8%)
Stage 3	12 (13.5%)
Stage 4	16 (18.0%)
Inflammatory activity^†^	
Grade 0	0 (0%)
Grade 1	22 (24.7%)
Grade 2	47 (52.8%)
Grade 3	13 (14.6%)
Grade 4	7 (7.9%)
Steatosis, n (%)	
None to mild	59 (66.3%)
Moderate	18 (20.2%)
Severe	12 (13.5%)
Laboratory findings^*^	
AST (IU/L)	65.1 ± 40.7
ALT (IU/L)	52.7 ± 37.8
Platelet (/10^6^)	215.5 ± 88.6
Bilirubin (mg/dL)	1.17 ± 1.01
Creatinine (mg/dL)	0.8 ± 0.7
PT (INR)	1.01 ± 0.11
Albumin (g/dL)	3.9 ± 0.5
Non-invasive values^*^	
MV flow, score	2.0 ± 1.0
MV flow, %	60.7 ± 22.1
RS85, kPa	9.4 ± 5.2
TE (kPa)	13.3 ± 13.0
CAP (dB/m)	262.7 ± 59.2

* Values were described as mean ± SD

† Histopathological results for liver fibrosis stage and inflammatory activity were evaluated according to the Batts-Ludwig Staging System scoring system

SD = standard deviation, HBV = hepatitis B virus, MASLD = Metabolic dysfunction-associated steatotic liver disease, ARLD = Alcohol-related liver disease, HCV = hepatitis C virus, AIH = Autoimmune hepatitis, PBC = Primary biliary cirrhosis, AST = aspartate transaminase, ALT = alanine transaminase, PT = prothrombin time, TE = transient elastography.

The distribution of liver fibrosis stages among the patients was as follows: 10 patients (11.2%) were at stage 0, 28 patients (31.5%) were at stage 1, 23 patients (25.8%) were at stage 2, 12 patients (13.5%) were at stage 3, and 16 patients (18.0%) were at stage 4. The mean MV grading score was 2.0 ± 1.0 points, and the mean percentage of flow was 60.7 ± 22.1. Of the patients included in the analysis, 86 underwent 2D-SWE simultaneously, and 83 underwent TE within a three-month period. The mean LS values by 2D-SWE and TE were 9.4 ± 5.2 kPa and 13.3 ± 13.0 kPa, respectively.

### Interobserver agreements of microvascular flow measurements

**Fig 2:**
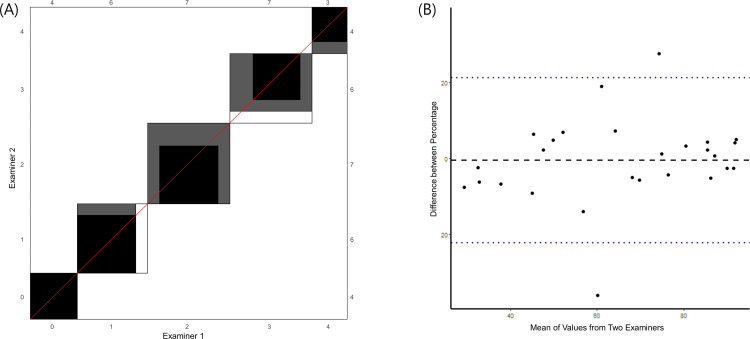
Interobserver agreement of microvascular flow (MV-flow) imaging for (a) scoring system and (b) percentage value.

For 27 of the study participants, two measurements of MVF were made by two different examiners to verify the consistency of the measurements. The Cohen’s κ coefficient of MV structure scoring system 0.718 (95% CI, 0.509–0.927, p < 0.001). ICC for percentage of MV flow was 0.867 (95% CI, 0.730–0.937, p < 0.001) (Fig 2).

### Correlation of liver histology with non-invasive tools

The MV-flow score displayed a strong positive correlation with the Batts-Ludwig Staging System (*r* = 0.751, *p* < 0.001). The percentage of MV-flow exhibited a negative correlation with the histologic staging of liver fibrosis (*r* = -0.494, *p* < 0.001). Additionally, the mean microvascular grading score showed positive correlations with other non-invasive markers such as TE (*r* = 0.774, *p* < 0.001) and 2D-SWE (*r* = 0.799, *p* < 0.001). The mean percentage of microvascular structure negatively correlated with TE (*r* = -0.571, p < 0.001) and 2D-SWE (*r* = -0.622, *p* < 0.001). The correlations between non-invasive tools are summarized in Table 3.

**Table 3:** Correlation analysis between non-invasive values.

	2D-SWE	MV-flow score	MV-flow percent	Pathologic staging	TE
**2D-SWE**		0.799 p < 0.001	−0.622 *p* < 0.001	0.749 *p* < 0.001	0.886 *p* < 0.001
**MV-flow score**	0.799 p < 0.001		−0.708 *p* < 0.001	0.751 *p* < 0.001	0.774 *p* < 0.001
**MV-flow percent**	−0.622 *p* < 0.001	−0.708 *p* < 0.001		−0.494 *p* < 0.001	−0.571 *p* < 0.001
**Pathologic staging**	0.748 *p* < 0.001	0.751 *p* < 0.001	−0.494 *p* < 0.001in		0.756 *p* < 0.001
**TE**	0.886 *p* < 0.001	0.774 *p* < 0.001	−0.571 *p* < 0.001	0.756 *p* < 0.001	

MV-flow = microvascular flow, 2D-SWE = two dimensional shearwave elastography, TE = transient elastography

### Diagnostic performance of single non-invasive tests for distinguishing liver fibrosis

**Fig 3:**
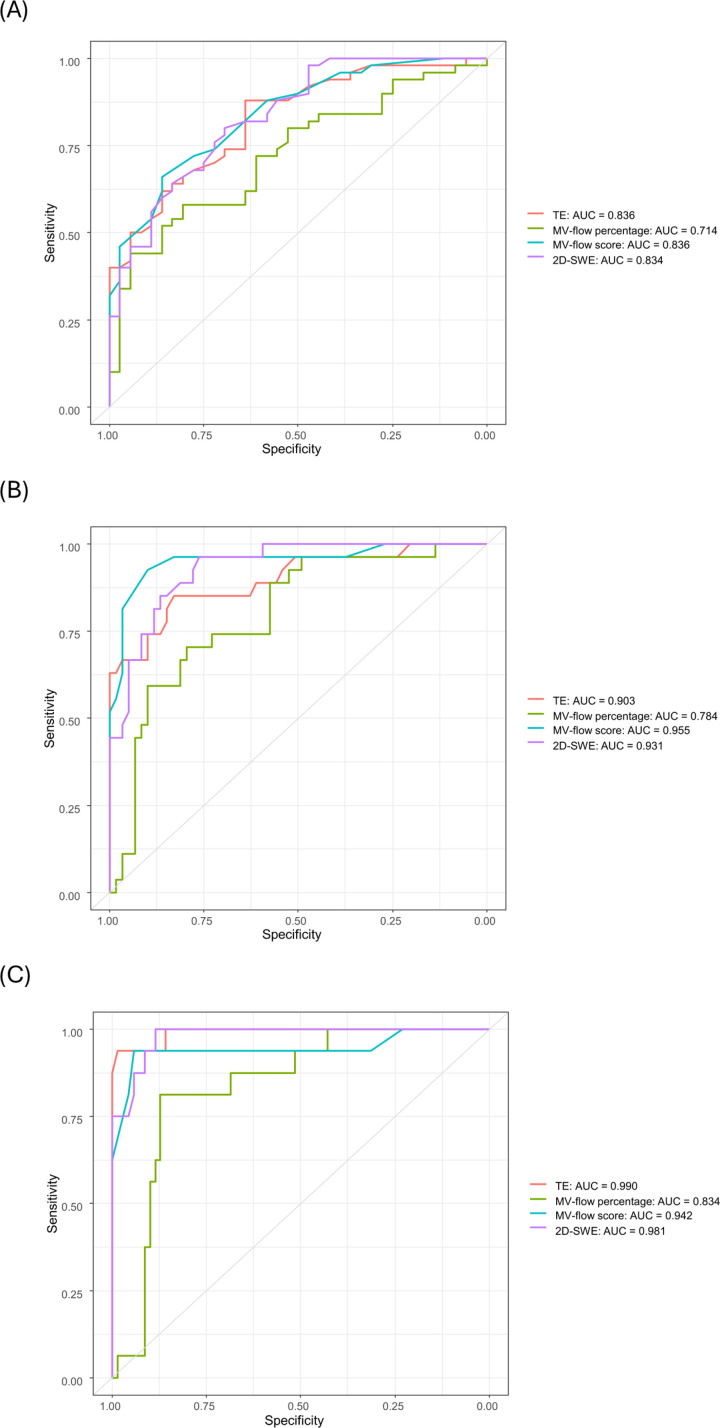
Receiver operating characteristic (ROC) curve of noninvasive tools for the diagnosis of (a) significant fibrosis (stage 2), (b) advanced fibrosis (stage 3), and (c) cirrhosis (stage 4).

ROC curves for identifying significant fibrosis (≥stage 2), advanced fibrosis (≥stage 3), and cirrhosis (stage 4) are depicted in Fig 3. The diagnostic performance, in terms of AUROC, of different non-invasive tools for distinguishing these liver fibrosis stages, along with their optimal cut-off values, is presented in Table 4.

**Table 4:** Diagnostic accuracy and optimal cutoff values of non-invasive value for the diagnosis of liver fibrosis.

Fibrosis Stage	≥stage 2 (95% CI)	≥stage 3 (95% CI)	stage 4 (95% CI)
**MV-flow, score (n = 89)**			
**Cutoff, points**	**2.1**	**2.5**	**2.9**
AUROC	0.836 (0.744-0.906)	0.955 (0.901-0.994)	0.942 (0.841-0.997)
Sensitivity, %	66.7	92.9	93.8
Specificity, %	84.2	90.2	93.2
PPV, %	85.0	81.3	75.0
NPV, %	65.3	96.5	98.6
Accuracy, %	74.2	91.0	93.3
**MV-flow, % (n = 89)**			
**Cutoff**	**53.7**	**51.5**	**43.1**
AUROC	0.714 (0.615-0.794)	0.784 (0.685-0.881)	0.834 (0.764-0.908)
Sensitivity, %	56.9	67.9	81.3
Specificity, %	78.9	78.7	86.3
PPV, %	78.4	59.4	56.5
NPV, %	57.7	84.2	95.5
Accuracy, %	66.3	75.3	85.4
**2D-SWE (n = 78)**			
**Cutoff, kPa**	**6.95**	**7.75**	**9.95**
AUROC	0.834 (0.729-0.919)	0.931 (0.875-0.974)	0.981 (0.944-0.996)
Sensitivity, %	80.0	96.3	100
Specificity, %	69.4	76.2	88.6
PPV, %	78.4	65.0	66.7
NPV, %	71.4	97.8	100
Accuracy, %	75.6	82.6	90.7
**TE (n = 83)**			
**Cutoff, kPa**	**7.30**	**9.45**	**16.5**
AUROC	0.836 (0.752-0.895)	0.903 (0.831-0.968)	0.990 (0.973-1.000)
Sensitivity, %	88.2	85.7	93.8
Specificity, %	65.8	83.6	98.6
PPV, %	77.6	70.6	93.8
NPV, %	80.7	92.7	98.6
Accuracy, %	78.7	84.3	97.8

AUROC = Area under ROC curve analysis, CI = Confidence Interval, PPV = Positive predictive value, NPV = Negative predictive value, TE = transient elastography.

Using the MV scoring system, the AUROCs for predicting ≥stage 2, ≥ stage 3, and cirrhosis were 0.836 (95% CI, 0.744–0.906), 0.955 (95% CI, 0.901–0.994), and 0.942 (95% CI, 0.841–0.997), respectively. The optimal cut-off values for ≥stage 2, ≥ stage 3, and cirrhosis using the MV scoring system were 2.1, 2.5, and 2.9, respectively. The AUROC exhibited its lowest value when considering the percentage of MV-flow. The AUROC for predicting ≥stage 2, ≥ stage 3, and cirrhosis based on the percentage of MV-flow was 0.714 (95% CI, 0.615–0.794), 0.784 (95% CI, 0.685–0.881), and 0.834 (95% CI, 0.764–0.908), with optimal cut-off values of 53.7%, 51.5%, and 43.1%, respectively.

### Comparison of diagnostic performance between non-invasive tools

The mean MV-flow imaging score exhibited the highest diagnostic accuracy among the evaluated non-invasive values for classifying ≥stage 2 and ≥stage 3. However, differences in AUROCs were not statistically significant, except for the percentage of MV-flow. For distinguishing cirrhosis, TE (*p* = 0.190) and 2D-SWE (*p* = 0.426) displayed higher AUROCs but showed no statistically significant differences compared to the MV scoring system. Notably, the percentage of MV-flow exhibited the lowest diagnostic performance compared to all other methods for classifying cirrhosis, ≥ stage 3, and ≥stage 2, with statistically significant differences in AUROCs (Table 5).

**Table 5:** Comparison of diagnostic performance between non-invasive values.

	AUC1	AUC2	*p*-value
Stage 2			
MV-flow score vs. MV-flow percent	0.837	0.714	**0.008**
MV-flow score vs. 2D-SWE	0.839	0.834	0.890
MV-flow score vs. TE	0.837	0.836	0.983
MV-flow percent vs. 2D-SWE	0.725	0.834	**0.035**
MV-flow percent vs. TE	0.714	0.836	**0.036**
2D-SWE vs. TE	0.834	0.845	0.764
Stage 3			
MV-flow score vs. MV-flow percent	0.955	0.784	**<0.001**
MV-flow score vs. 2D-SWE	0.954	0.932	0.456
MV-flow score vs. TE	0.955	0.903	0.170
MV-flow percent vs. 2D-SWE	0.797	0.932	**0.006**
MV-flow percent vs. TE	0.784	0.903	**0.046**
2D-SWE vs. TE	0.932	0.897	0.172
Stage 4			
MV-flow score vs. MV-flow percent	0.942	0.834	**0.017**
MV-flow score vs. 2D-SWE	0.944	0.981	0.426
MV-flow score vs. TE	0.942	0.990	0.190
MV-flow percent vs. 2D-SWE	0.837	0.981	**<0.001**
MV-flow percent vs. TE	0.834	0.990	**0.001**
2D-SWE vs. TE	0.981	0.991	0.544

AUC = Area under curve, MV-flow = microvascular flow, 2D-SWE = two dimensional shearwave elastography, TE = transient elastography.

### Combination of non-invasive tools for the diagnosis of advanced liver fibrosis

**Fig 4:**
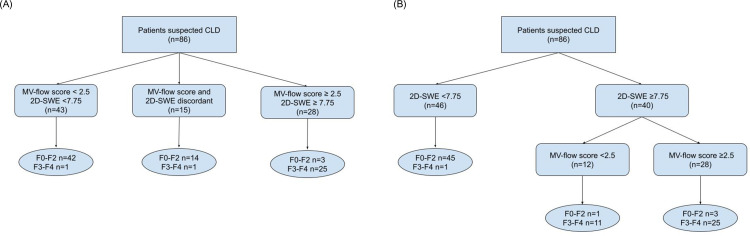
Accuracy of combination of microvascular scoring system with liver stiffness (LS) by 2D-SWE for predicting advanced fibrosis. (a) paired combination (b) sequential combination. CLD, Chronic liver disease; MV-flow, microvascular flow; 2D-SWE, 2-dimensional shear wave elastography.

Statistical analysis revealed that the mean value of the MV structure scoring system showed the highest AUROC and accuracy in predicting advanced fibrosis (≥stage 3). However, 8 out of 89 patients (9.0%) were misclassified based on the MV structure scoring system. 2D-SWE, which can be measured concurrently with the scoring system using the same equipment, exhibited the second-highest AUROC. However, if it were used to diagnose stage 3, 15 patients (17.4%) would be misclassified. To reduce such misclassifications, a paired combination of MV score with 2D-SWE was evaluated, which reduced the likelihood of patients being misclassified to 4/86 (4.7%), lower than when either tool was used individually. The likelihood of missing advanced fibrosis in individuals with an MV score below 2.5 and a 2D-SWE below 7.75 was 1 in 86 (1.2%). To address discrepancies between the MV score and the 2D-SWE, a serial combination approach was applied. Initially, patients were classified using 2D-SWE, which demonstrated a higher NPV, and then reclassified based on their MV scores. This diagnostic approach for advanced fibrosis resulted in 5 patients (5.8%) being misclassified, with one case (1.2%) of advanced fibrosis missed. This classification method is detailed in Fig 4.

## Discussion

In this study, we observed a strong correlation between the MV-flow scoring system and the Batts-Ludwig Staging System. Furthermore, the percentage of MV-flow imaging displayed a negative correlation with histologic staging of liver fibrosis, while the mean microvascular grading score exhibited a positive correlation with other non-invasive markers such as TE and 2D-SWE. In the ROC curve analysis, the MV-flow scoring system demonstrated high AUROC values for predicting significant fibrosis (≥stage 2), advanced fibrosis (≥stage 3), and cirrhosis (stage 4). We also provided optimal cut-off values for each of these stages. The present study supports the effectiveness of combining the MV-flow score and 2D-SWE to prevent patient misclassification, thus providing reliable information regarding signs of advanced liver fibrosis.

Previous studies investigating hepatic angiography have indicated a correlation between the progression of chronic liver disease and observed phenomena, such as tortuous vessels, tapered vascular structures, inconsistent branching patterns, and grouped branch formations [21,22]. In situations where hepatic angiography’s invasive nature poses challenges, attempts have been made to explore the association between liver fibrosis and the tortuosity or blunting of microvessels using Superb Microvascular Imaging [9,16–18]. MV-flow imaging, as a Doppler method, offers heightened sensitivity to slower flow rates without the interference of motion artifacts, distinguishing it from other Doppler imaging techniques [23].

The findings of this study consistently align with previous research that assessed microvessel imaging, 2D-SWE, and TE. In a study by Koyama et al. (2016) [9], MV-flow imaging was proposed as a potentially valuable marker for reflecting liver fibrosis. Moreover, in their study of 100 Hepatitis C patients, Kuroda et al. (2016) [18], found a strong correlation with pathologically confirmed liver fibrosis stages. Balik et al. (2019) [17] reported a high AUC value for severe fibrosis using superb microvascular imaging technology at 0.881.

Prior studies [9,16–18] investigating the utility of microvascular imaging in assessing liver fibrosis primarily focused on predicting advanced fibrosis (stage 3) or cirrhosis. In contrast, the present study evaluated not only advanced fibrosis and cirrhosis but also significant fibrosis (stage 2). It demonstrated equivalent performance to TE or 2D-SWE. To overcome the limitations of semi-quantitative staging in predicting the degree of liver fibrosis, we averaged results from five measurements. Based on this, we established cut-off values corresponding to stage 2, stage 3, and stage 4.

To date, superb microvascular imaging has proven effective in assessing thyroid nodules [24], breast masses [25], and focal liver lesions [26], but there have been limited data exploring its role in evaluating the extent of liver fibrosis. Our study used Samsung Medison’s microvascular flow imaging equipment to evaluate tissue, specifically to measure the severity of fibrosis in chronic liver disease. The outcomes are consistent with microvessel imaging studies conducted by other manufacturers (Canon Medical Systems [16,17], Toshiba Medical Systems [9,18]), which reported similar findings previously. Moreover, despite its lower diagnostic utility compared to the MV-flow scoring system, we made an attempt at quantitative analysis by assessing the percentage of MV-flow imaging, a novel approach not previously undertaken in research.

We conducted a comprehensive assessment of various non-invasive tools for evaluating liver fibrosis. This study not only compared individual tools like the MV scoring system, TE, and 2D-SWE but also explored their correlations with one another and with histologic staging. We illustrated the utility of the MV scoring system in distinguishing significant fibrosis, advanced fibrosis, and cirrhosis in chronic liver disease. Moreover, the diagnostic accuracy of the MV scoring system was found to be on par with other widely utilized non-invasive methods.

The elastographic system may face challenges in obtaining values from patients with physical limitations like ascites, as elastic waves don’t propagate well through fluids [27]. However, since MV-flow imaging assesses liver fibrosis based on structural changes, it could potentially offer advantages in evaluating such patients. These two tests could complement each other’s limitations and provide a more integrated interpretation.

With the equipment we employed, it is feasible to simultaneously perform both 2D-SWE and the MV scoring system during a single ultrasound examination. This not only enhances patient convenience by eliminating the need for multiple examinations but also enables a more accurate diagnosis of liver conditions in a single session. This integrated approach not only improves patient comfort but also fosters a more precise understanding of the patient’s liver status through a unified evaluation. As we have proposed, we anticipate that combining these two non-invasive assessments will facilitate more accurate diagnoses of patients’ conditions.

The present study has several limitations that should be acknowledged. Firstly, the relatively small number of participants and heterogenous etiologies limits the generalizability of our findings and may not fully represent a larger population with diverse characteristics. Future studies with larger and more homogeneous cohorts are essential to validate these preliminary findings and refine the utility of MV-flow imaging as a diagnostic tool for liver fibrosis. Secondly, the absence of a validation cohort prevents us from confirming and replicating our findings in an independent sample, which is essential to ensure the robustness and reliability of our results. Thirdly, although inter-observer variability in the MV-flow scoring system was observed, it was only detectable in a small number of cases. Fourthly, this study was conducted during the COVID-19 pandemic (2020–2022), a period which may have influenced participant recruitment and clinical workflows. While no specific adjustments were made to account for pandemic-related factors, it is possible that access to healthcare services or patient demographics during this time differed from non-pandemic periods. Finally, although this study focused on evaluating the diagnostic performance of MV-flow imaging in patients with chronic liver disease, a control group of healthy individuals or patients with non-fibrotic liver conditions could provide valuable comparisons.

In conclusion, our findings suggest that MV-Flow is an effective non-invasive method for predicting the stage of liver fibrosis in patients with chronic liver disease. MV-Flow demonstrates comparability to other established non-invasive diagnostic methods. When combined with 2D-SWE measurements, which utilize the same equipment and can be conducted simultaneously, it exhibits better performance in identifying patients who could potentially avoid a liver biopsy. Further large-scale studies are needed to validate and build upon our findings.

## Supporting information

S1 File Data. (XLSX)

## Abbreviations

CLD: chronic liver disease
MV-flow: microvascular flow
TE: transient elastography
2D-SWE: two-dimensional shear wave elastography
AUROC: area under the receiver operating characteristic curve
LS: liver stiffness
PPV: positive predictive value
NPV: negative predictive value
SD: standard deviation
ROC: receiver-operating characteristic
CI: confidence interval.
