# Correction: Diagnostic performance of microvascular flow imaging for noninvasive assessment of liver fibrosis in chronic liver disease

**DOI:** 10.1371/journal.pone.0335628

**Published:** 2025-10-28

**Authors:** Hae Won Yoo, Chan Jin Yang, Jeong-Ju Yoo, Young Chang, Sae Hwan Lee, Soung Won Jeong, Jae Young Jang, Gab Jin Cheon, Young Seok Kim, Hong Soo Kim, Sang Gyune Kim

Fig 4 is incorrect. The authors have provided a corrected version here.

**Fig 4:**
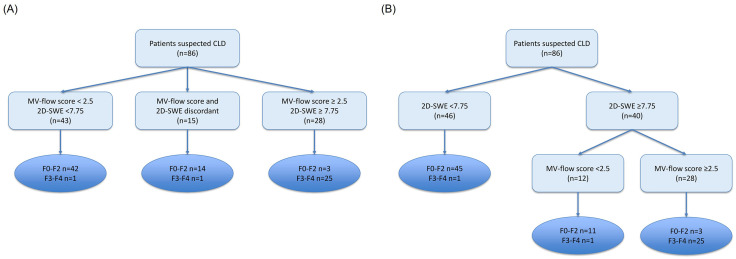
Accuracy of combination of microvascular scoring system with liver stiffness (LS) by 2D-SWE for predicting advanced fibrosis. (a) paired combination (b) sequential combination. CLD, Chronic liver disease; MV-flow, microvascular flow; 2D-SWE, 2-dimensional shear wave elastography.
